# Accounting for Forest Carbon Loss from Natural Gas Development in a Major U.S. Gas Region

**DOI:** 10.1021/acs.est.6c00986

**Published:** 2026-09-01

**Authors:** Elliot S. Shannon, Andrew O. Finley, Paul B. May, Grant M. Domke, Jane R. Foster

**Affiliations:** † Department of Forestry, 3078 Michigan State University, East Lansing, Michigan 48824, United States; ‡ Department of Statistics and Probability, 3078 Michigan State University, East Lansing, Michigan 48824, United States; § Department of Electrical Engineering and Computer Science, 6806 South Dakota School of Mines & Technology, Rapid City, South Dakota 57701, United States; ∥ Northern Research Station, United States Department of Agriculture Forest Service, St. Paul, Minnesota 55108, United States; ⊥ Southern Research Station, United States Department of Agriculture Forest Service, Knoxville, Tennessee 37920, United States

**Keywords:** energy development, forest carbon, nationwide forest inventory, small area estimation, spatiotemporal, Bayesian

## Abstract

Natural gas development in forested landscapes can cause substantial forest disturbance, yet its impacts on forest carbon remain poorly quantified. In the Appalachian Basin of the United States, where natural gas production is expected to continue expanding in the coming decades, understanding these impacts is important for evaluating trade-offs between energy production and forest ecosystem services. Here, we combine nationwide forest inventory and remotely sensed data within a Bayesian spatiotemporal modeling framework to quantify forest disturbance, carbon loss, and opportunity cost associated with natural gas development between 2008 and 2021. Using permitted well locations in Ohio, Pennsylvania, and West Virginia, we generate pixel-level estimates with associated uncertainty and aggregate results to characterize regional patterns. We estimate that 10 854 ha of forest land were disturbed, resulting in 542 675 (±4275) Mg of forest carbon loss and an opportunity cost of 575 246 (±30 774) Mg of carbon (equivalent to the annual emissions of approximately 464 000 and 492 000 gasoline-powered passenger vehicles, respectively). Disturbance rates were greatest in northern West Virginia, while opportunity costs were highest in northeastern Pennsylvania. To our knowledge, this study provides the first regional-scale quantification of forest carbon loss associated with natural gas development in the Appalachian Basin. The results demonstrate the importance of accounting for forest carbon impacts when evaluating energy development and provide information needed to support future mitigation, planning, and optimization efforts.

## Graphical abstract

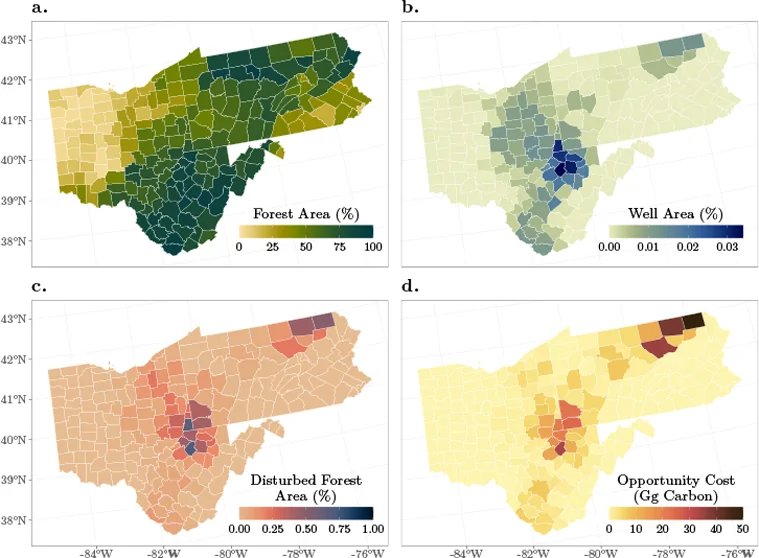

## 1 Introduction

Natural gas energy production in the United States (U.S.) has accelerated in recent years to meet rising energy demands. Associated land use changes, including those on forest lands, are an important consideration, as studies suggest that energy production will be the primary driver of land use change in the U.S. through 2040.^1,2^ The U.S. states of Ohio, Pennsylvania, and West Virginia, which encompass much of the Appalachian Basin and its Marcellus and Utica shale formations (Figure 1), are major natural gas producers, with output expected to increase in the coming decades.^3^ Natural gas extraction from the Marcellus and Utica shale formations has a long history in the region,^4,5^ reaching record production levels in 2021, when the region accounted for about one-third of the nation’s dry natural gas output.^6,7^ Much of the region is also heavily forested, providing important environmental and economic benefits.

**Figure 1:**
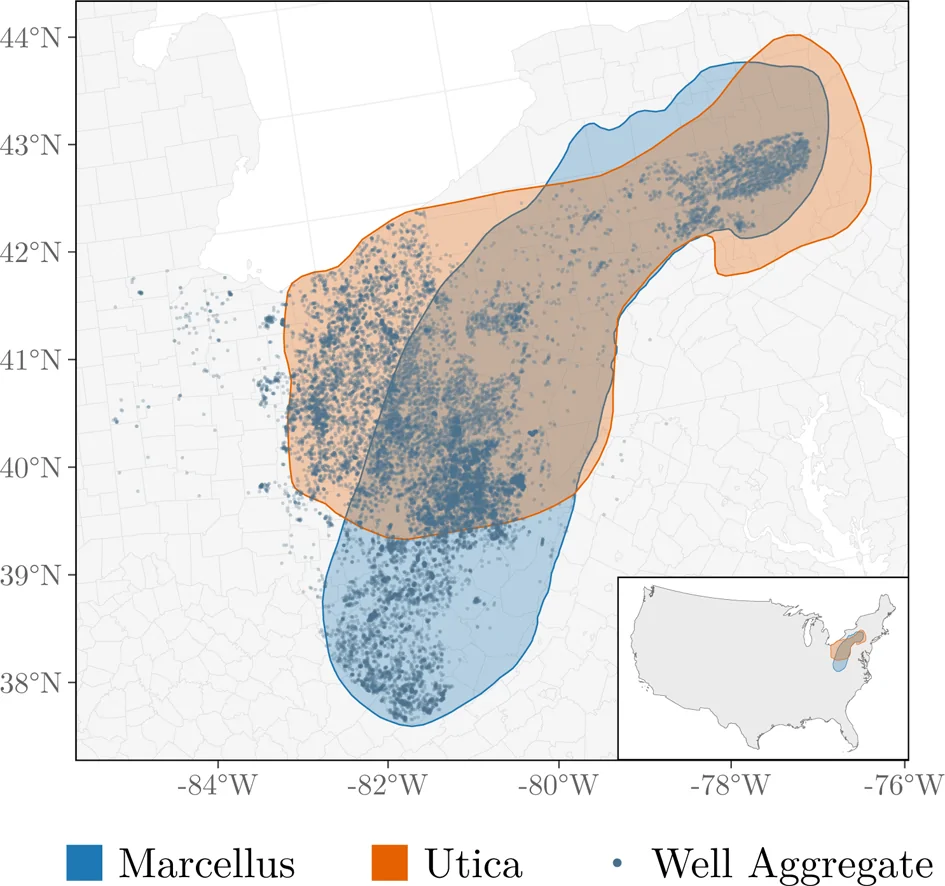
Well aggregates within the U.S. states of Ohio, Pennsylvania, and West Virginia, displayed with the Marcellus and Utica shale formation boundaries. An inset map displays the well aggregates and shale formation locations within the CONUS.

Natural gas development requires extensive supporting infrastructure, including well pads, roads, compressor stations, and gathering pipelines. In forested landscapes, such infrastructure can alter forest ecosystems through direct land conversion and fragmentation.^8^ Numerous studies have documented forest loss, habitat fragmentation, and canopy disturbance associated with natural gas development across the Appalachian Basin.^4,9–15^ These studies consistently identify well pads, access roads, gathering pipelines, and other supporting infrastructure as drivers of landscape change and core forest loss. The documented impacts of natural gas development have motivated efforts to reduce environmental costs through improved planning and infrastructure placement. Previous studies have evaluated mitigation practices, such as locating well pads along the margins of core forest areas and collocating pipelines with existing infrastructure to reduce fragmentation.^11,16^ Optimization frameworks have been developed to explicitly evaluate trade-offs between infrastructure costs and environmental impacts.^17,18^ Collectively, this body of work demonstrates that environmental impacts can be reduced through more systematic development planning, provided those impacts can be effectively quantified.

Despite substantial progress in characterizing and mitigating landscape impacts, the consequences of natural gas development for forest carbon remain poorly understood. Forests represent one of the largest terrestrial carbon sinks in the U.S. and play a critical role in climate mitigation efforts.^19,20^ While previous studies have quantified land cover change, habitat fragmentation, and canopy disturbance associated with natural gas infrastructure, to our knowledge, no study has quantified regional-scale forest carbon loss attributable to natural gas development in the Appalachian Basin. Carbon impacts represent an important but largely unquantified component of the environmental trade-offs considered in mitigation and optimization frameworks. Quantifying these losses is necessary to fully evaluate the environmental consequences of energy development and to support mitigation, planning, and optimization efforts that seek to reduce the impacts on forest ecosystems.

Recent advances in spatiotemporal forest carbon modeling now enable estimation of forest carbon dynamics over large spatial and temporal domains using national forest inventory and remotely sensed data.^21–23^ In particular, small area estimation (SAE) methods have been developed to leverage sparse inventory measurements and auxiliary data to produce spatially explicit estimates of forest carbon status and change while quantifying uncertainty. Recent contiguous U.S. (CONUS)-scale SAE analyses identified areas of significant forest carbon loss across portions of the Appalachian Basin between 2008 and 2021.^22,23^ Several of these areas coincide with regions of extensive natural gas development, motivating a more direct assessment of the relationship between natural gas development and forest carbon loss. Here, we address this gap by quantifying forest carbon impacts associated with natural gas development across Ohio, Pennsylvania, and West Virginia from 2008 to 2021.

To meet this objective, we developed a Bayesian spatiotemporal small area estimation framework using forest inventory and analysis (FIA) measurements, remotely sensed tree canopy cover (TCC) data, and spatial records of natural gas development to estimate forest carbon density at high spatial and temporal resolution. The framework provides direct uncertainty quantification and enables estimation of disturbance extent, carbon loss, and opportunity cost, where opportunity cost includes both carbon removed through disturbance and foregone future carbon accumulation. Specifically, within permitted natural gas well areas, we estimate (i) the extent of forest disturbance, (ii) the associated forest carbon loss, (iii) the opportunity cost of disturbance, and (iv) the spatial variation in disturbance and carbon impacts across counties in the region.

## 2 Materials and Methods

### 2.1 Study Area and Well Data

The U.S. states of Ohio, Pennsylvania, and West Virginia encompass much of the Appalachian Basin and its Marcellus and Utica shale formations. Significant natural gas development has occurred throughout the region in recent decades, resulting in a dense network of well pads and supporting infrastructure. All publicly available permitted natural gas well locations within these states between 2008 and 2021 were obtained from the Ohio Department of Natural Resources, the Pennsylvania Department of Environmental Protection, and the West Virginia Department of Environmental Protection.

Following estimated disturbance areas associated with natural gas well pads reported by Grushecky et al.,^15^ circular buffers were generated around each reported well permit location, with areas of 4.7, 6.2, and 4.4 ha for locations in Ohio, Pennsylvania, and West Virginia, respectively. Overlapping buffer regions were then merged, resulting in 19 111 aggregated well permit areas ranging from 4.4 to 815.6 ha in size (hereafter referred to as “well aggregates”; Figure 1). The study area was then defined as a 10 km buffer surrounding the convex hull of the well aggregates, which was used in subsequent model fitting described in sections 2.2 and 2.3.

### 2.2 Forest Data

The FIA program is tasked with the design and collection of NFI data across the U.S., whereby a network of approximately 300 000 inventory plots are visited on a rotating basis every 5–10 years.^24,25^ Forest variables of interest, including derived quantities, such as live forest carbon density (Mg/ha) (simply referred to as “forest carbon” in the remaining text), are measured at inventory plots and serve as primary data for subsequent SAE models. Additionally, FIA determines whether each inventory plot measurement occurs on forested or non-forestland, and this information can be used to build Bernoulli models of forest presence or absence. In this study, 99 345 FIA plot measurements within the study area (Figure 1) were used to predict the presence of forest in Bernoulli models between 2008 and 2021, while a subset of 56 881 forested FIA plot measurements were used to estimate forest carbon conditional on forest presence.

Remotely sensed TCC data are produced by the USDA Forest Service as part of the National Land Cover Database (NLCD), which represents the percentage of land area covered by forest canopy.^26^ The TCC data are derived from multispectral Landsat observations and are available as annual raster images with a 30 m pixel resolution across the CONUS between 2008 and 2021. Here, pixel-level TCC values coincident with FIA plot measurements and well aggregates were used as covariates to inform estimates of forest presence and forest carbon. Additionally, TCC values for the entire study region were used to predict baseline estimates of forest area and forest carbon from fitted models.

### 2.3 Model

We used the latent Gaussian model (LGM) proposed by ref 21 to estimate pixel-level forest carbon within well aggregates. Let *y*(**s**, *t*) ≥ 0 be the forest carbon at location **s** at time *t*. To accommodate the significant probability mass occurring at zero in the forest carbon data model, a binary process *p*(**s**, *t*) is defined where *p*(**s**, *t*) = 1 ⇔ *y*(**s**, *t*) > 0. The LGM is then

$$p\left(s,t\right)\overset{iid}{∼}\mathrm{Bernoulli}\left(\mu_{p}\left(s,t\right)\right)$$ 1

$$\mathrm{logit}\left(\mu_{p}\left(s,t\right)\right)=\eta_{p}+x\left(s,t\right)\beta_{p}$$ 2

where η_*p*_ is an intercept term and β_*p*_ is a regression coefficient corresponding to TCC, denoted *x*(**s**, *t*). The forest carbon *y*(**s**, *t*) is then modeled as

$$y\left(s,t\right)=\eta_{y}+x\left(s,t\right)\beta_{y}+w_{y}\left(s,t\right)+\varepsilon_{y}\left(s,t\right)$$ 3

where $\varepsilon_{y}\left(s,t\right)\overset{iid}{∼}\mathrm{Normal}\left(0,\tau^{2}\right)$ is a normally distributed random error term, η_*y*_ is an intercept term, β_*y*_ is a regression coefficient corresponding to *x*(**s**, *t*), and *w*_*y*_(**s**, *t*) is a spatiotemporal random intercept following a mean zero Gaussian process. A final equation for forest carbon is then written as *y*(**s**, *t*) × *p*(**s**, *t*), which we denote as *z*(**s**, *t*).

A Gaussian process specification for *w*_*y*_(**s**, *t*) is represented as a sum of independent component Gaussian processes as in ref 21. These components allow for process variation to be represented at different scales. Here, we specify two components for the process, allowing for both short- and long-range dependencies in the spatial and temporal domain. Specifically, for a generic mean zero spatiotemporal Gaussian process *w*(**s**, *t*), we write *w*(**s**, *t*) ∼ *GP*(0, *C*(·, ·)), where *C*(δ_s_, δ_t_) is a covariance function depending on the spatial and temporal lags δ_s_ and δ_t_, respectively. Implementing the dual component covariance structure as proposed in ref 21, we have

$$C\left(\delta_{s},\delta_{t}\right)=\sigma^{2}\left(\alpha e^{-\delta_{s}\phi_{1}-\delta_{t}\lambda_{1}}+\left(1-\alpha \right)e^{-\delta_{s}\phi_{2}-\delta_{t}\lambda_{2}}\right)$$ 4

where ϕ_1_ > 0 and λ_1_ > 0 are the short-range spatial and temporal range parameters and ϕ_2_ > 0 and λ_2_ > 0 are the long-range spatial and temporal range parameters, respectively. Further, σ^2^ > 0 is a spatiotemporal variance parameter, and 0 < α < 1. Hence, the full model for *z*(**s**, *t*) includes parameters **θ** = [η_*p*_, β_*p*_, η_*y*_, β_*y*_, σ_*y*_^2^, α_*y*_, ϕ_*y*,1_, ϕ_*y*,2_, λ_*y*,1_, λ_*y*,2_, τ^2^]^T^.

### 2.4 Disturbance

Predictions of forest carbon *z*(**s**, *t*), as well as model components *p*(**s**, *t*) and *y*(**s**, *t*), are readily available for pixels within well aggregates and are obtained via their posterior predictive distributions. To understand baseline levels of forest area across the entire study domain, *p*(**s**, *t*) was estimated at each TCC pixel location in the first year (*t* = 1). For a given area, such as a county, with geographic area denoted as S , the total forest area is determined as $\underset{s\in S}{\sum }p\left(s,1\right)$ , which is used as a baseline to quantify percent forest area disturbed for each county.

To assess instances of predicted forest disturbance at a location **s**, threshold criteria are implemented based on estimated values of μ_*p*_(**s**, *t*). Specifically, for location **s** and time *t*, the probability of disturbance is calculated as *d*(**s**, *t*) = μ_*p*_(**s**, *t* – 1) × (1 – μ_*p*_(**s**, *t*)), which represents the probability that location **s** was forested at time *t* – 1 and not forested at time *t*. A location **s** is determined to have been disturbed if *d*(**s**, *t*) > 0.5 for some *t* > 1. The selection of this threshold seeks to balance identification of marginal disturbances associated with natural gas development while filtering minor disturbances associated with natural forest processes.

### 2.5 Opportunity Cost

To assess opportunity costs of disturbed well aggregate areas, an autoregressive moving average (ARMA) model is fit using TCC values from undisturbed well areas to estimate carbon values for disturbed pixels. Autoregressive models have been used previously to model forest structure dynamics and have demonstrated value in forecasting over short to intermediate temporal scales.^27^ Specifically, TCC values from 10 000 undisturbed locations were used to fit the ARMA model, which was then used to predict undisturbed TCC values *x̃*(**s**, *t*) for disturbed locations **s** for all *t* > 1. These predicted TCC values *x̃*(**s**, *t*) represent the undisturbed scenario for location **s**, which is used to calculate *z̃*(**s**, *t*) following models described in section 2.3 using *x̃*(**s**, *t*) in place of *x*(**s**, *t*). Since the exact time of disturbance is not known, the opportunity cost for disturbed location **s** is taken as the difference in disturbed and undisturbed forest carbon in the final year and is calculated as *z̃*(**s**, *T*) – *z*(**s**, *T*).

## 3 Results

The model detailed in section 2.3 was fit using FIA and TCC data described in section 2.2 to estimate pixel-level forest carbon for well aggregate locations outlined in section 2.1 (Figure 1). Threshold criteria described in section 2.4 were applied to identify disturbed pixels. Opportunity costs were estimated for disturbed pixel locations following methods outlined in section 2.5. Additionally, to inform estimates of quantities of interest, including percent forest area disturbed, baseline estimates of forest area and carbon were generated from models fit by using pixel-level TCC data for the entire study region.

Following disturbance threshold criteria described in section 2.4, an estimated 10 854 ha of forest land was disturbed between 2009 and 2021, resulting in 542 675 Mg of forest carbon loss (534 296 and 551 054 lower and upper 95% credible interval limits). This disturbed area represents approximately 0.07% of the total forested area within the states of Ohio, Pennsylvania, and West Virginia. The opportunity cost in these disturbed areas amounted to 575 246 Mg of carbon (514 928 and 635 563).

Pixel-level carbon estimates were generated for all well aggregates, which are shown for a single well in Figure 2. National Agriculture Imagery Program (NAIP) imagery for disturbed well aggregates is regularly available over the time period and highlights the progression of natural gas development. Annual estimates of total carbon for each well aggregate are compared for both disturbed and undisturbed scenarios, which provide insight into the opportunity cost associated with natural gas development.

**Figure 2:**
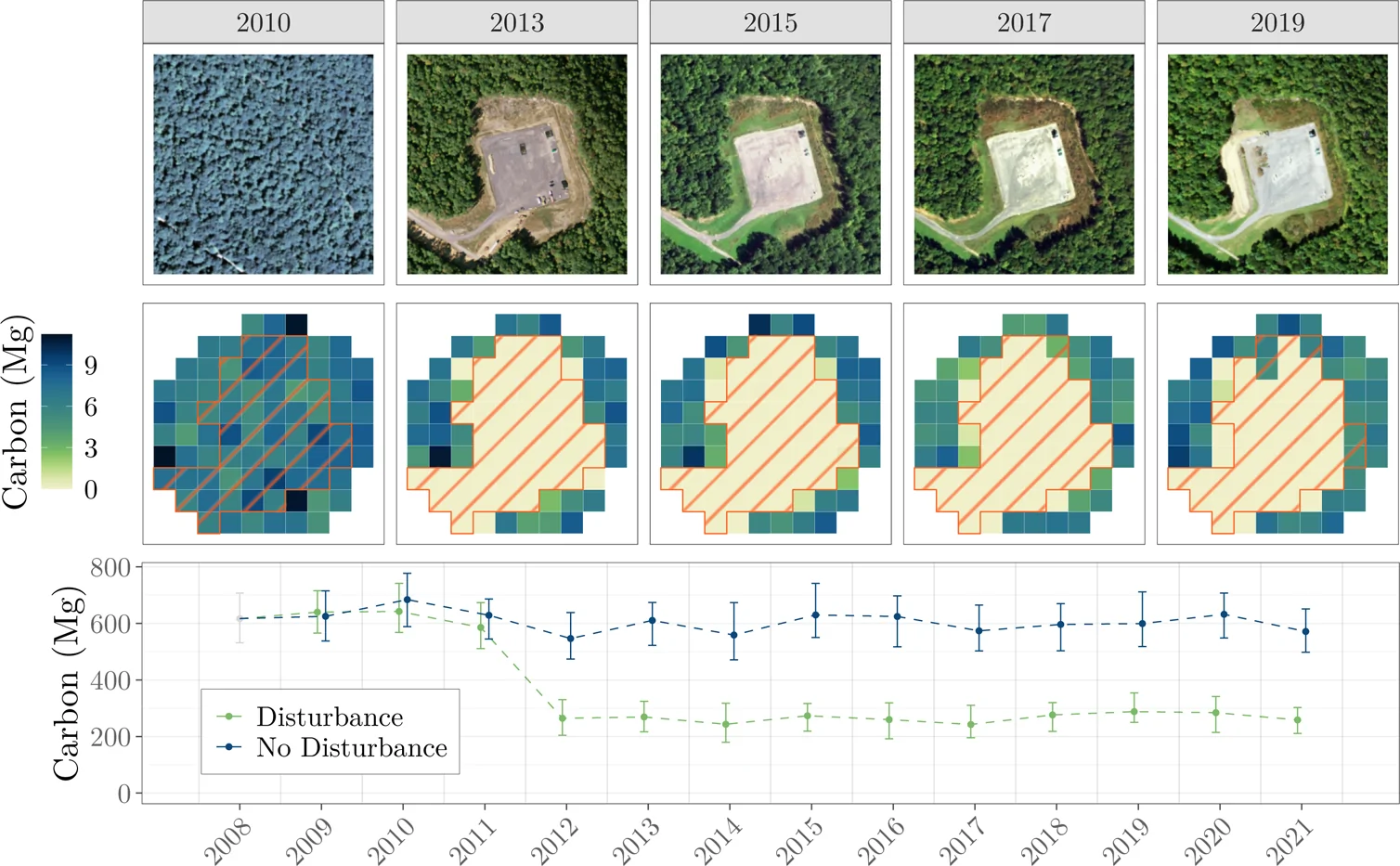
(Top row) NAIP imagery displaying the time series progression of natural gas development for a well site in Tioga County, Pennsylvania. (Center row) Posterior median pixel-level carbon estimates (*z*(**s**, *t*)) for years corresponding to available NAIP imagery. Disturbed pixels are denoted with an orange stripe pattern. (Bottom row) Posterior median and 95% credible intervals of total carbon at this well site for both disturbed and undisturbed scenarios.

Pixel-level quantities and estimates are aggregated to the county level and are displayed in Figure 3. The percent forest area is shown in Figure 3a, which highlights the distribution of forested areas in the region. The percent of total county area comprised of well aggregates is displayed in Figure 3b, which visualizes the distribution of natural gas development in the region. Insights from Figures 3a and b support patterns of percent forest area disturbed shown in Figure 3c, where heavily forested counties associated with significant natural gas development experience the highest levels of forest disturbance. Doddridge County, West Virginia, exhibited the highest disturbance levels, amounting to approximately 0.7% (521 ha) of the total forest land in the county. Opportunity costs associated with disturbed locations are aggregated to the county level and displayed in Figure 3d. Here, total opportunity costs are highest for the large, heavily forested counties in northeastern Pennsylvania, with Susquehanna County, Pennsylvania, exhibiting the greatest total opportunity cost of 49 255 Mg of carbon (46 193 and 52 317).

**Figure 3:**
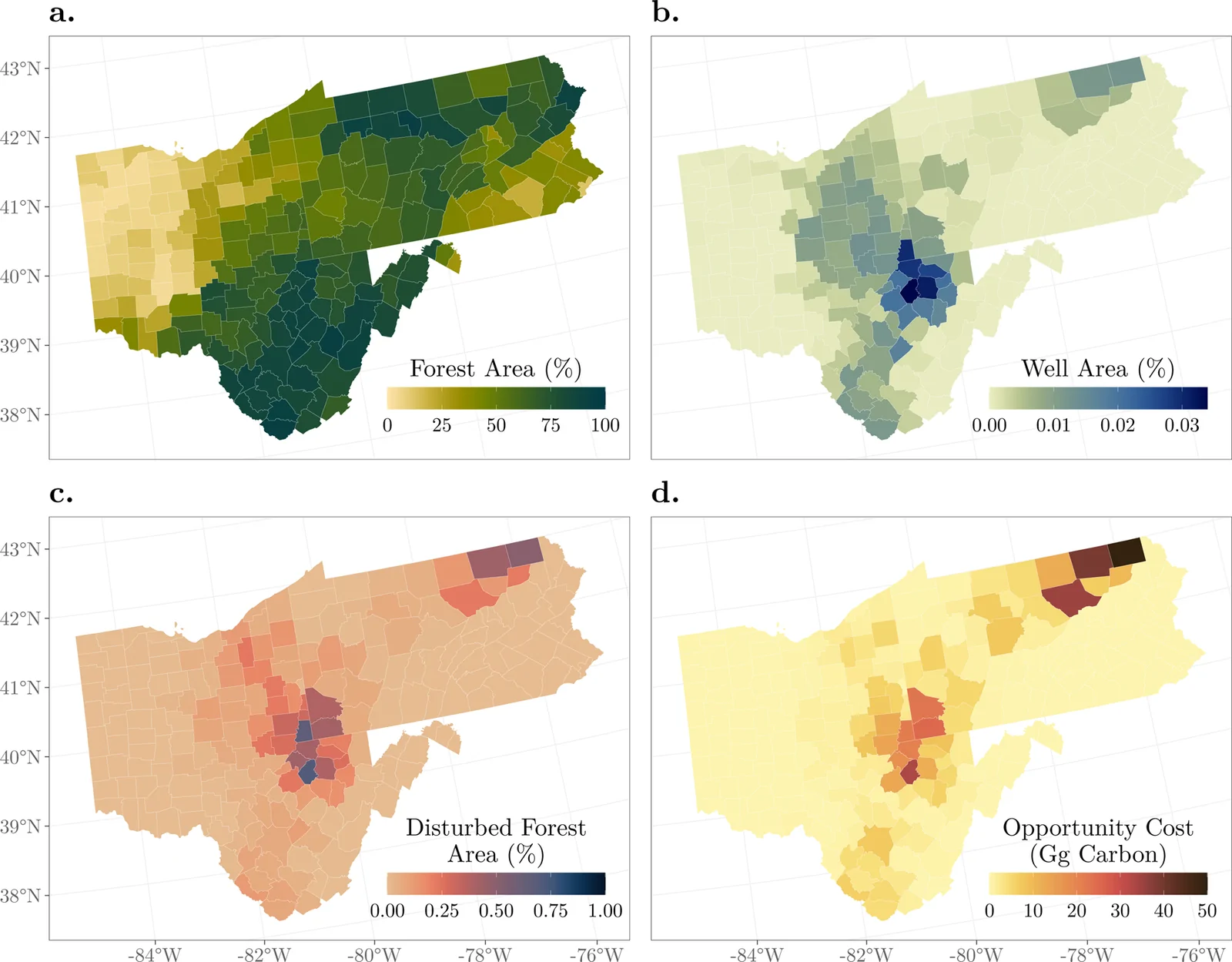
(a) Posterior median estimates of percent forest area for counties in Ohio, Pennsylvania, and West Virginia. (b) County-level percent well area, representing the percent of the total county area occupied by well aggregate areas. (c) Posterior median estimates of percent forest area disturbed at the county level. (d) Posterior median total opportunity costs estimated at the county level.

Baseline pixel-level carbon estimates for Susquehanna County, Pennsylvania, along with opportunity costs at disturbed locations, are displayed in Figure 4. Estimates shown in Figure 4a highlight the complex distribution of forests in the county, and the focal area shown in Figure 4b provides insight into the density and impact of natural gas development in the area through pixel-level opportunity cost estimates.

**Figure 4:**
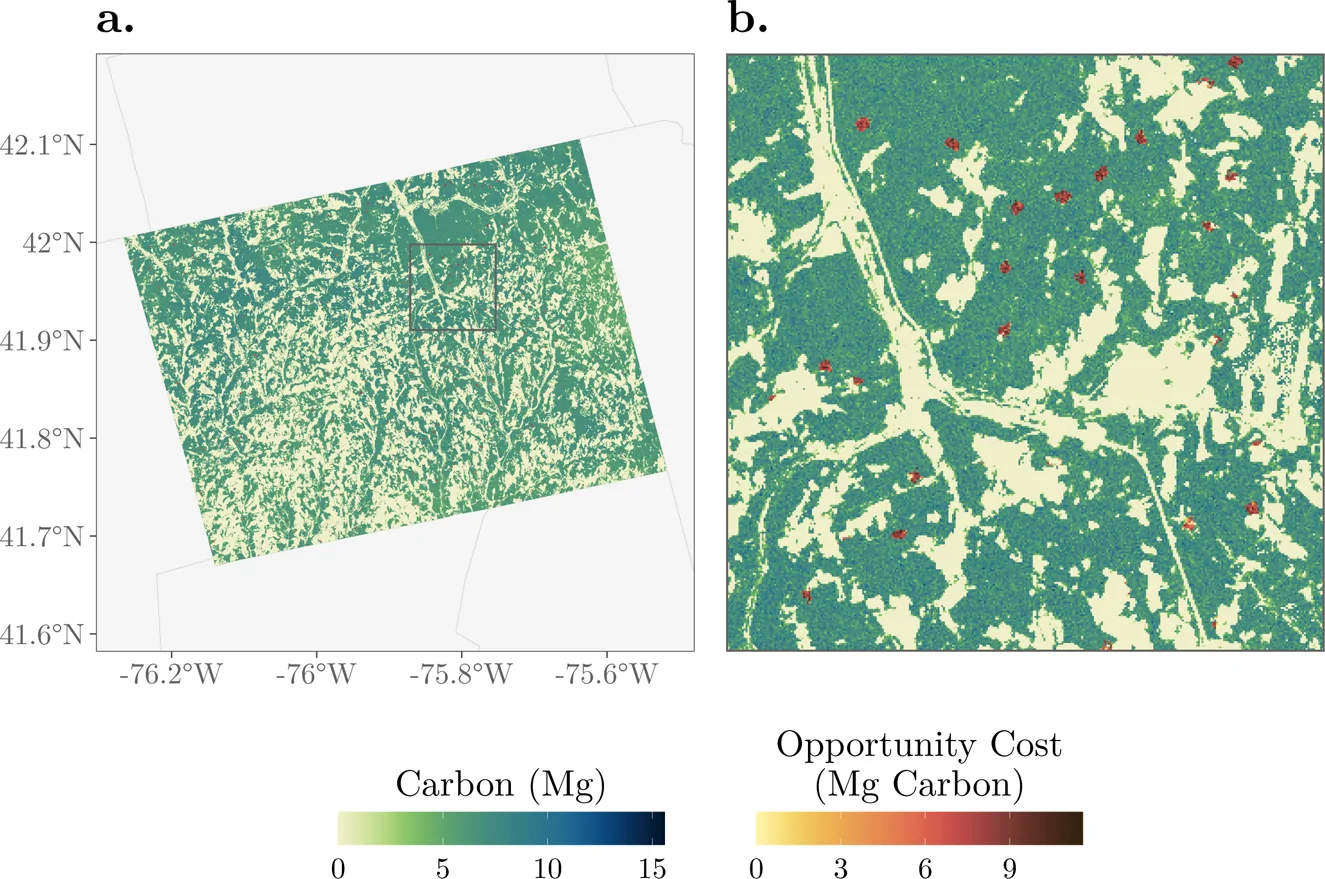
(a) Baseline pixel-level posterior median forest carbon estimates for Susquehanna County, Pennsylvania, with posterior median opportunity costs mapped for disturbed pixel locations. (b) Focal area highlighting the fine distribution patterns of forest carbon in the county as well as the density and magnitude of opportunity costs at disturbed pixel locations.

## 4 Discussion

This study provides, to our knowledge, the first regional-scale assessment of forest carbon loss associated with natural gas development in the Appalachian Basin. Using NFI and remotely sensed data within a Bayesian spatiotemporal SAE framework, we quantified disturbance extent, forest carbon loss, and opportunity cost associated with permitted natural gas development across Ohio, Pennsylvania, and West Virginia between 2008 and 2021. Estimates produced at the pixel level and aggregated to counties revealed substantial spatial variation in the magnitude and distribution of impacts across the region.

Overall, an estimated 10 854 ha of forest land was disturbed between 2009 and 2021. This value is lower than the 23 000 ha of affected land reported by Grushecky et al.^15^ for oil and natural gas development in the region between 2007 and 2017. However, the two estimates were not directly comparable. The analysis of Grushecky et al.^15^ considered all land-cover classification changes within buffered development areas, whereas the approach presented here estimates pixel-level probabilities of forest presence using NFI and remotely sensed TCC data. Consequently, our estimates reflect changes in forest structure rather than land-cover classifications alone. As outlined in section 2.4, estimates of disturbed area are sensitive to the selected disturbance threshold and therefore should be interpreted as a conservative estimate of forest loss associated with natural gas development. At the county level, the highest rates of disturbance occurred in eastern Ohio, northern West Virginia, and northeastern Pennsylvania. These patterns generally correspond to areas characterized by both extensive forest cover and intensive natural gas development, suggesting that the interaction between baseline forest conditions and development intensity is an important determinant of forest impacts.

Within disturbed well aggregate locations, we estimate that approximately 542 675 Mg of forest carbon was removed, contributing to an opportunity cost of 575 246 Mg of carbon. Following the U.S. Environmental Protection Agency,^28^ these values are equivalent to the annual greenhouse gas emissions from approximately 464 133 and 491 990 gasoline-powered passenger vehicles, respectively. Although these losses represent a relatively small fraction of total regional forest carbon stocks, they constitute a measurable environmental cost of energy development concentrated within areas directly affected by infrastructure expansion. Previous studies have documented substantial canopy removal associated with oil and gas development in the region;^14^ the results presented here extend those findings by translating disturbance into estimates of forest carbon loss and foregone carbon accumulation. Opportunity cost captures both carbon directly removed through disturbance and carbon that would have accumulated if disturbance had not occurred. As illustrated in Figure 2, these foregone gains can be substantial even when direct disturbance footprints are relatively small, highlighting the importance of considering both immediate and longer term impacts when evaluating forest carbon consequences of development.

While the current study demonstrates a novel and statistically rigorous framework for quantifying forest carbon impacts associated with natural gas development, opportunities remain for refinement and future work. First, the analysis relies on permitted natural gas well locations and disturbance extents derived from buffered well areas rather than verified infrastructure footprints. Consequently, the estimated carbon losses and opportunity costs should be interpreted as impacts associated with permitted well aggregate locations and not as causal effects attributable solely to well-pad construction. In many cases, observed carbon loss within buffer areas was associated with roads, gathering pipelines, and other supporting infrastructure rather than with well pads alone. Future work could improve attribution by integrating high-resolution imagery and machine learning approaches to identify and classify specific infrastructure features and disturbance footprints.^29^ Second, the disturbance criteria used here identify areas experiencing forest loss but do not explicitly estimate the timing of individual disturbance events, limiting our ability to assess the temporal patterns of development. Methods based on time-series analysis and change-point detection could be used to associate disturbance events with specific years and development activities.^30,31^ Finally, opportunity costs were estimated by using a relatively simple autoregressive model to represent forest conditions in the absence of disturbance. More sophisticated models of forest growth and succession could improve estimates of counterfactual carbon accumulation and provide a more complete assessment of long-term opportunity costs.^27^

The impacts of natural gas development in forested regions are substantial, and methods to quantify their magnitude and spatial distribution are essential for balancing rising energy demands with the maintenance of forest ecosystem services.^9^ In the context of climate change, forests provide important carbon sequestration benefits, while energy development can reduce carbon storage through forest disturbances and loss. As a result, decisions regarding the placement and design of energy infrastructure involve trade-offs among energy production, habitat conservation, and carbon storage. Previous studies have shown that environmental impacts of natural gas development can be reduced through improved infrastructure placement and optimization-based planning approaches.^11,17^ However, these frameworks have largely relied on habitat, fragmentation, or land use metrics and have not explicitly incorporated forest carbon impacts. By providing spatially explicit estimates of disturbance extent, carbon loss, and opportunity cost with associated uncertainty, the framework developed here supplies a critical component for future mitigation, planning, and optimization efforts. More broadly, integrating forest inventory, remote sensing, and statistical modeling provides a pathway for evaluating trade-offs between energy development and forest ecosystem services across multiple spatial scales, supporting future decision-making in both energy and forest management.
